# A Case Report of Necrotizing Neutrophilic Dermatosis: A Sheep in Wolf’s Clothing

**DOI:** 10.7759/cureus.26498

**Published:** 2022-07-01

**Authors:** Devon Ackerman, Chandat Phan, Marcos Kuroki, Matthew Helm, Nimalan A Jeganathan

**Affiliations:** 1 College of Medicine, Penn State Health Milton S. Hershey Medical Center, Hershey, USA; 2 General Surgery, Penn State Health Milton S. Hershey Medical Center, Hershey, USA; 3 Dermatology, Penn State Health Milton S. Hershey Medical Center, Hershey, USA; 4 Colorectal Surgery, Penn State Health Milton S. Hershey Medical Center, Hershey, USA

**Keywords:** surgery, sweet's syndrome, pyoderma gangrenosum, pathergy, necrotizing soft tissue infection, necrotizing neutrophilic dermatosis

## Abstract

Neutrophilic dermatosis (ND) is a category of diseases characterized by trauma-induced, autoinflammatory cutaneous eruption. Comorbid systemic disease is common with a predilection for malignancy, inflammatory bowel disease, and rheumatologic disease. Rarely, it can manifest with aseptic shock, an entity referred to as necrotizing neutrophilic dermatosis (NND). NND may occur in the postoperative setting and is often misdiagnosed as a necrotizing soft tissue infection. Unfortunately, the treatment for a necrotizing soft tissue infection, namely, wide debridement, is often detrimental in the setting of NND. We present the case of a woman with underlying myelodysplastic syndrome who developed episodic postoperative hemodynamic collapse followed by delayed necrotic peristomal ulceration following colonic diversion for complicated diverticulitis. Infectious workup and operative re-exploration were unrevealing. Pathologic assessment of affected skin tissue showed changes consistent with ND, ultimately leading to the diagnosis of NND. Her clinical course dramatically improved with the initiation of immunosuppressive therapy. The mimicry of NND to a potentially lethal necrotizing soft-tissue infection creates a grave diagnostic dilemma in the postoperative period. A general lack of knowledge of NND among non-dermatologic specialists produces an opportunity for misdiagnosis and inappropriate surgical interventions, namely, serial debridement. Several clinical cues may aid in the earlier recognition of NND. The cornerstone of treatment involves systemic corticosteroid therapy with adjunctive therapy for refractory cases. NND must be considered in the differential diagnosis of necrotizing soft tissue infection as early recognition may result in the avoidance of deleterious surgical interventions.

## Introduction

Neutrophilic dermatoses (NDs) comprise a spectrum of cutaneous disorders characterized by non-infectious neutrophilic infiltration of dermal tissue and associated autoinflammation [1,2]. Although incompletely understood, aberrant cytokine and chemokine regulation is believed to play a central role in pathogenesis [1,3]. NDs are commonly associated with concomitant inflammatory disease and have a predilection for pathergy, an exaggerated response to minor trauma [1,2]. Clinically, they may manifest as a variety of lesions, including ulcers, plaques, bullae, pustules, papules, nodules, or abscesses [1]. The underlying inflammatory process is generally responsive to immunosuppressive therapy [1,2]. Necrotizing neutrophilic dermatosis (NND) represents the emerging term given to the subset of ND cases accompanied by severe systemic inflammation and accompanying shock [3]. The alternate differential diagnosis for an erythematous and/or painful rash in conjunction with systemic toxicity is a necrotizing soft tissue infection (NSTI), a highly lethal condition requiring emergent and often extensive surgical debridement to prevent certain mortality [4]. Given the rapid progression shared by both NND and NSTI along with their markedly divergent management strategies, parsing out the correct diagnosis is of paramount importance for clinicians [3,5,6].

There is a lack of awareness of NDs in non-dermatological fields. In a recent study of 20 major surgical and emergency medicine textbooks, only 10 mentioned pyoderma gangrenosum (PG), a common subtype of ND, and none included PG within the provided NSTI differential [7]. The aim of this report is to help bridge the knowledge gap. Here, we present the case of a 66-year-old female who underwent a diverting colostomy for perforated diverticulitis complicated by the development of postoperative NND at an academic, tertiary care center. This work has been reported in line with the Surgical CAse REport (SCARE) criteria [8].

## Case presentation

**Figure 1:**
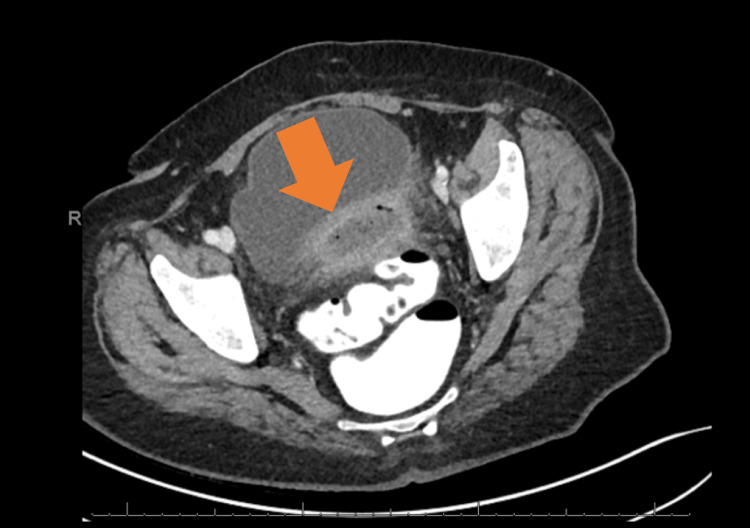
Preoperative CT scan demonstrating thick-walled, mildly enhancing complex fluid collection anterior to the sigmoid colon consistent with a perisigmoid abscess of diverticular origin. CT: computed tomography

A 66-year-old female with a recent diagnosis of myelodysplastic syndrome (MDS) on azacitidine presented to our emergency room with a three-day history of abdominal pain and decreased appetite. Two days prior, she had been discharged on oral antibiotics from another hospital with a diagnosis of complicated diverticulitis. A radiographic review of her prior computed tomography (CT) scan demonstrated a small pericolic abscess consistent with complicated (Hinchey 1b) sigmoid diverticulitis. Her abscess was not amenable to percutaneous drainage. After several days in the hospital, she was discharged home with oral antibiotics and plans for outpatient follow-up with interval colonoscopy. However, four days later, she again presented with worsening left lower quadrant abdominal pain and sudden passage of stool per vagina. She had previously undergone a hysterectomy and speculum examination which confirmed feculent drainage at the vaginal cuff consistent with a colovaginal fistula. The findings were confirmed by the CT scan (Figure 1).

Further laboratory workup demonstrated pancytopenia with a white blood cell count of 3.5 K/µL, hemoglobin of 9.4 g/dL, and platelet count of 37 K/µL. Both the need to deliver future chemotherapy for her MDS and failure of non-operative therapy led us to conclude that surgical intervention would be beneficial. Given the patient’s inability to tolerate a major resection in her debilitated state, palliative fecal diversion with a laparoscopic-assisted loop transverse colostomy was performed. In the immediate postoperative period, she did well with minimal pain, resumption of a normal diet with good colostomy function, and cessation of feculent vaginal drainage.

**Figure 2:**
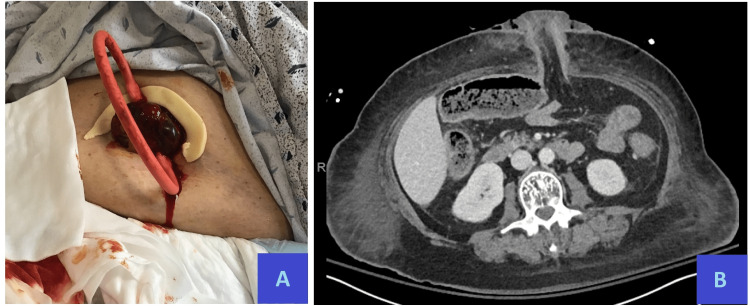
(A) Postoperative day three, loop colostomy with venous congestion and contact bleeding but normal appearance of peristomal skin. Additionally pictured, tan-colored skin barrier ring and red rubber catheter stoma bar. (B) Postoperative day four, CT scan showing left upper quadrant loop colostomy with non-specific subcutaneous edema. CT: computed tomography

Due to transfusion requirements related to MDS disease severity, she received two transfusions of packed red blood cells and multiple platelet transfusions to maintain hemoglobin of 7 g/dL and platelets of 10 K/µL. On postoperative day (POD) two, the patient developed a fever of 38.8°C with rigors, tachycardia, and hypoxemia immediately following transfusion of red blood cells. Immediate hemolysis testing was negative. Given the rapid clinical improvement with acetaminophen and diphenhydramine, a febrile non-hemolytic transfusion reaction was suspected. A very similar episode occurred on POD four except that she developed hypotension refractory to fluid resuscitation. On physical examination, the abdomen was soft and mildly tender, consistent with postoperative inflammation. The colostomy appeared edematous, which was unchanged from the day prior (Figure 2A). A bedside assessment for colonic ischemia at the level of the fascia (i.e., “test-tube test”) was negative [9]. Due to continued clinical deterioration, a CT scan of the abdomen was obtained. The findings demonstrated a decrease in the size of the perisigmoid abscess and mild body wall edema in the peristomal region (Figure 2B). However, there was no evidence of an intra-abdominal source of sepsis.

Over the next three days, she continued to have episodic high-grade fevers (>40.0°C), severe lactic acidosis (>9.5 mmol/L), and hypotension requiring vasopressor support. In consultation with the Infectious Disease team, the patient was treated empirically with broad-spectrum antibiotics for presumed sepsis of unknown etiology. Most curiously, the occurrences were episodic and short-lived with the restoration of normal vital signs between the episodes. Throughout, the patient’s only complaint was generalized fatigue.

**Figure 3:**
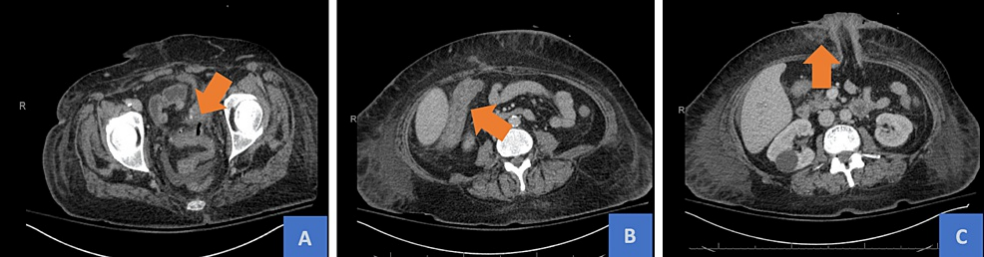
(A) Interval improvement of perisigmoid fluid collection after fecal diversion. (B) Wall thickening with associated fat stranding of the ascending and proximal transverse colon concerning for colitis. (C) Persistent body wall edema without subcutaneous emphysema.

On POD eight, she developed worsening hemodynamic deterioration and an acutely painful, erythematous rash around the colostomy. Another repeat abdominal CT scan was obtained to rule out an intra-abdominal infectious source, demonstrating a decreased perisigmoid abscess and unchanged abdominal wall edema. However, new colonic thickening and adjacent fat stranding of the ascending colon raised concerns for possible ischemic colitis (Figure 3).

She was urgently brought to the operating room to evaluate for sepsis secondary to colonic ischemia and/or NSTI. Fortunately, initial endoscopic evaluation through both the afferent and efferent limbs of her colostomy revealed no evidence of mucosal ischemia. Furthermore, midline laparotomy with an examination of the small and large intestine confirmed no hollow viscus injury. Lastly, attention was directed to the new-onset cutaneous lesion. Inspection of the peristomal skin showed a large violaceous discoloration with multiple shallow ulcerations limited to the superficial layers. Palpation failed to reveal crepitus or fluctuance, and after very limited debridement, the underlying fascia was found to be healthy. The colostomy was re-sited due to the extensive peristomal ulceration, which would prevent effective stoma pouching. The patient returned to the intensive care unit intubated and in guarded condition. While the exact diagnosis was unknown, findings from this exploratory surgery effectively ruled out an intra-abdominal source of sepsis and NSTI.

**Figure 4:**
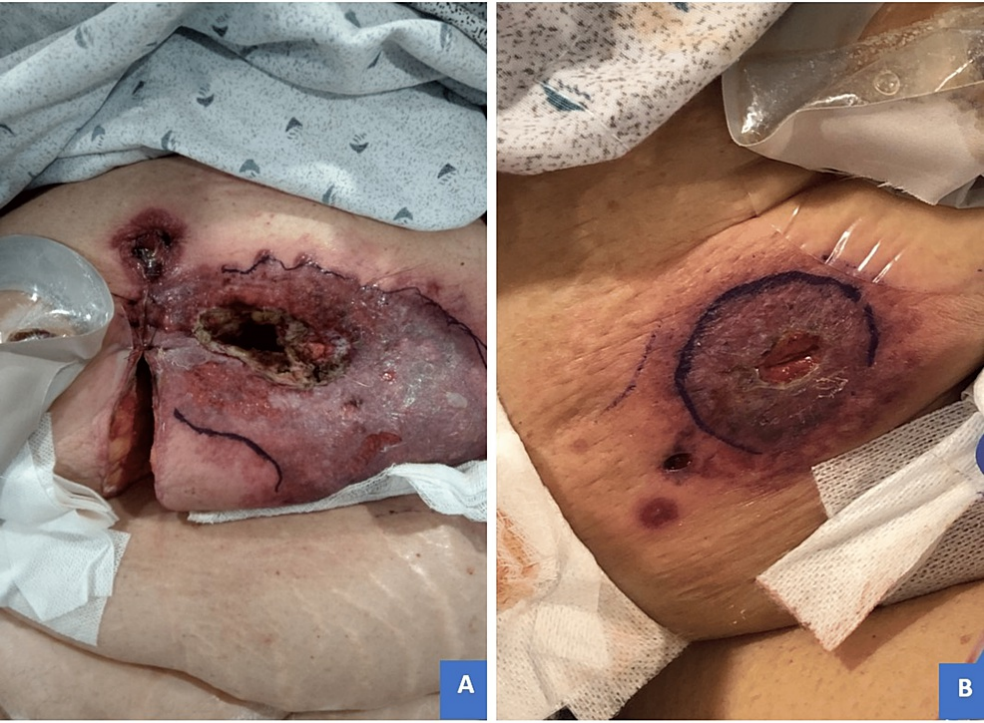
Pre-corticosteroid treatment imaging of cutaneous eruptions. (A) Peristomal region: confluent ulceration with central necrotic tissue overhanging demarcated violaceous border extending to and involving midline incision three days after re-exploration. (B) Laparoscopic port site: similar discoloration with central skin breakdown and characteristic satellite lesions at the port site.

Over the next 48 hours, the patient continued to be hemodynamically unstable despite supportive care and escalating empiric antimicrobial therapies. On physical examination, the original left-sided peristomal skin wound showed significant enlargement with confluent purple discoloration and sharp demarcation from adjacent skin (Figure 4A). She developed a similar rash at her laparoscopic port sites (Figure 4B), as well as her left inframammary crease and labia at the site of a pressure injury from her Foley catheter (not pictured).

**Figure 5:**
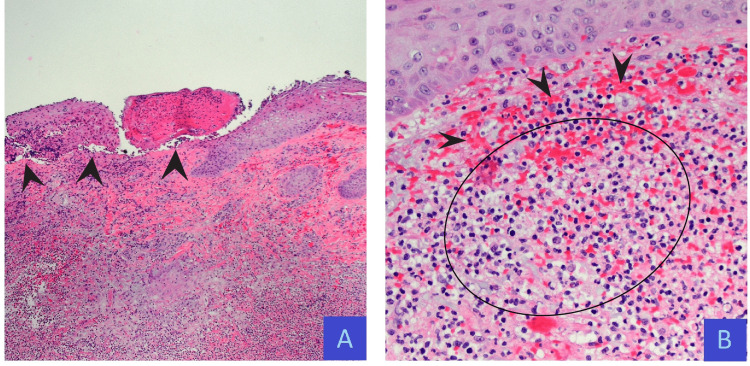
Right lower quadrant punch biopsy with negative infectious stains. (A) H&E at 4× magnification, necrotic epidermis (arrows) with underlying mixed dermal inflammation consisting of a neutrophil-predominant infiltrate. (B) H&E at 20× magnification, mixed dermal infiltrate composed of neutrophils, lymphocytes, histiocytes (circle), and extravasated red blood cells (arrows). H&E: hematoxylin and eosin

Given the lesion’s morphology and predilection for sites of trauma paired with the patient’s concomitant MDS, a primary dermatologic disorder was suspected. After consulting the Dermatology service, PG was confirmed by a punch biopsy. Histopathology findings demonstrated dermal necrosis with a sterile, dense neutrophil-predominant infiltrate (Figure 5).

**Figure 6:**
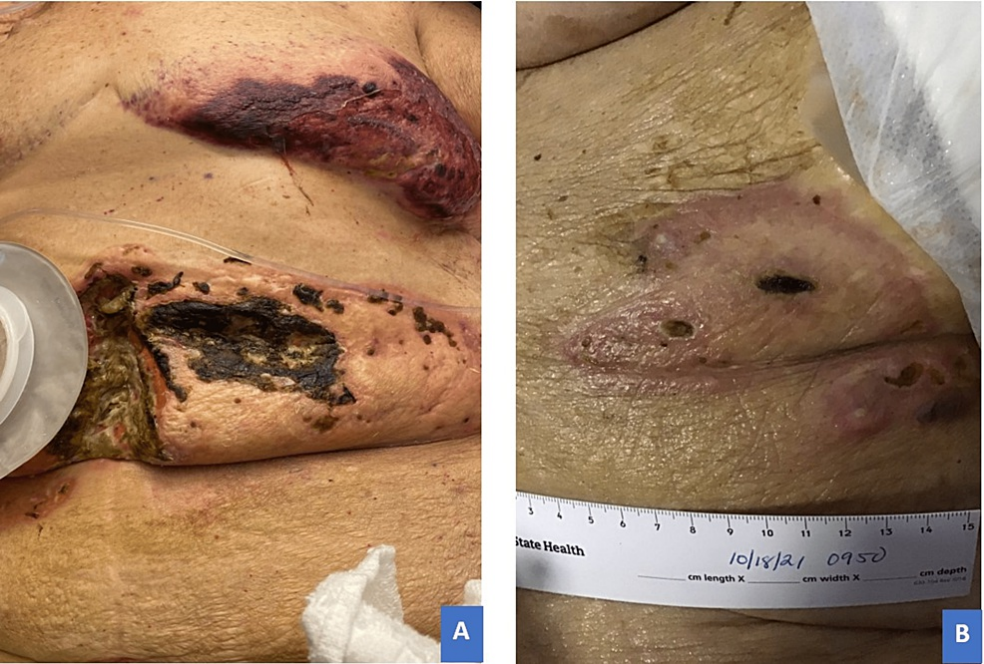
Post-corticosteroid treatment, day 28: dramatic improvement of cutaneous lesions showing border collapse, fading discoloration with central eschar, and skin dimpling/scarring. (A) Peristomal region. (B) Laparoscopic port site.

The combined findings of aseptic shock and PG ultimately led to the unifying diagnosis of NND. Intravenous methylprednisolone at a dose of 1 mg/kg/day was promptly initiated. Within 24 hours, she was weaned off vasopressor therapy and extubated. Due to expansion of the violaceous rim on both abdominal wounds, adjunctive 2 g/kg intravenous immunoglobulin was added to the regimen. The sentinel cutaneous lesions began to show the first signs of improvement by day four of treatment and continued to progress with ongoing therapy (Figure 6). Unfortunately, her pancytopenia failed to respond to ongoing transfusions, and the patient suffered considerable deconditioning. She left the hospital on day 71 of admission after electing for home hospice care.

## Discussion

Our patient’s case of NND closely aligns with the proposed diagnostic criteria of (1) clinicopathological evidence of ND, (2) systemic involvement manifesting as a shock, (3) negative microbiology workup, (4) pathergic response, and (5) rapid response to immunosuppressive therapy [3]. Multiple comorbid conditions, including malignancy, are associated with NND, highlighting our patient’s MDS as a likely risk factor [3,10,11]. Diverse yet distinct subtypes of NDs exist, ranging from Sweet’s syndrome, PG, and ND of the dorsal hand. Our patient’s presentation most closely resembled the PG subtype of NND which demonstrates ulcerative lesions with violaceous rimming and neutrophilic infiltration down to the hypodermis layer [1,3,10].

While our case highlights diagnostic and management challenges of NND, additional responsibility falls on surgeons to rapidly recognize the condition as unique from NSTI. This stems from the discrepancy in management between the two entities. Management of NSTI, namely, extensive surgical debridement, is not indicated in the setting of NND. Misdiagnosis and inappropriate surgical interventions have led to significant morbidity, including extremity amputation [3,11,12]. Although the two conditions overlap significantly in clinical presentation, the presence of extremely rapid progression (i.e., hours vs. days), crepitus, or murky drainage should immediately heighten the concern for NSTI, triggering surgical debridement (Table 1). When the prior findings are absent, a prompt biopsy of the ulcerated edge with rapid gram stain and histopathologic assessment is likely to provide the confirmatory testing for NND versus NSTI [3,11,13].

**Table 1:** Clinical characteristics for NND and NSTI. NND: necrotizing neutrophilic dermatosis; NSTI: necrotizing soft tissue infection [4,5,10,13-16].

Common features		
Cutaneous lesions: Erythema, necrosis, ecchymosis, bullae, induration		
Severe lesion pain		
May occur in the postoperative setting		
Systemic involvement: Fever, shock, leukocytosis		
Distinguishing features	NND	NSTI
Clinical progression	Days	Hours
Lesion characteristics	Violaceous, demarcated border, satellite lesions	Crepitus, “dishwater” drainage
Microbiology	Negative infectious tissue culture and staining	Positive infectious tissue culture and staining
Response to trauma	Pathergic	Improves with debridement
Response to systemic steroids	Improves condition	Possibly worsens condition

A study of NDs with aseptic shock found that the majority of reported cases demonstrated cutaneous eruption before systemic toxicity, sometimes months in advance [17]. Our patient’s course exhibited the opposite, with signs of presumed sepsis occurring nine days prior to the onset of peristomal erythema and ulceration. During this cutaneous latency period, a negative infectious workup and undulating pattern of shock suggested the etiology was likely non-infectious. A greater awareness of NND could have resulted in an earlier diagnosis and, thus, avoidance of unwarranted surgical interventions.

The mainstay of management of NND is with immunosuppression. Systemic corticosteroids alone or in combination with cyclosporine are considered first-line therapies [11]. Other corticosteroid-sparing agents and adjunctive therapies have been used with modest success (Table 2). Our patient’s cutaneous lesions responded well to the combination treatment of methylprednisolone and intravenous immunoglobulin.

**Table 2:** Pharmacologic treatment options for NND. NND: necrotizing neutrophilic dermatosis [11,17].

First-line	Systemic corticosteroids ± cyclosporine	
Adjunctive agents	Adalimumab, azathioprine, colchicine, cyclophosphamide, dapsone	Hyperbaric oxygen, infliximab, methotrexate, mycophenolate, thalidomide

## Conclusions

NND manifests as postoperative dermatologic lesions accompanied by shock, and therefore, poses clinical mimicry to a necrotizing soft tissue infection. To promptly choose appropriate management, a surgeon must at least suspect cutaneous autoinflammation from ND as opposed to infection. Although expedited dermatological consultation may be beneficial, increased awareness by surgeons of this alternative etiology through advancing surgical education curricula and literature exposure is of paramount importance.
